# Changes in Migration and Mortality Among Patients With Kidney Failure in Puerto Rico After Hurricane Maria

**DOI:** 10.1001/jamahealthforum.2022.2534

**Published:** 2022-08-12

**Authors:** Maricruz Rivera-Hernandez, Daeho Kim, Kevin H. Nguyen, Rebecca Thorsness, Yoojin Lee, Shailender Swaminathan, Rajnish Mehrotra, Amal N. Trivedi

**Affiliations:** 1 Department of Health Services, Policy, and Practice, Brown University School of Public Health, Providence, Rhode Island; 2 Providence Veterans Affairs Medical Center, Providence, Rhode Island; 3 Department of Medicine, University of Washington School of Medicine, Seattle

## Abstract

**Importance:**

On September 20, 2017, one of the most destructive hurricanes in US history made landfall in Puerto Rico. Anecdotal reports suggest that many persons with kidney failure left Puerto Rico after Hurricane Maria; however, empirical estimates of migration and health outcomes for this population are scarce.

**Objective:**

To assess the changes in migration and mortality among patients with kidney failure in need of dialysis treatment in Puerto Rico after Hurricane Maria.

**Design, Setting, and Participants:**

This cross-sectional study used an interrupted time-series design of 6-month mortality rates and migration of 11 652 patients who received hemodialysis or peritoneal dialysis care in Puerto Rico before Hurricane Maria (before October 1, 2017) and/or during and after Hurricane Maria (on/after October 1, 2017). Data analyses were performed from February 12, 2019, to June 16, 2022..

**Main Outcomes and Measures:**

Number of unique persons dialyzed in Puerto Rico per quarter; receipt of dialysis treatment outside Puerto Rico per quarter; and 6-month mortality rate per person-quarter for all persons undergoing dialysis.

**Exposures:**

Hurricane Maria.

**Results:**

The entire study sample comprised 11 652 unique persons (mean [SD] age, 59 [14.7] years; 7157 [61.6%] men and 4465 [38.4%] women; 10 675 [91.9%] Hispanic individuals). There were 9022 patients with kidney failure and dialysis treatment before and 5397 patients after Hurricane Maria. Before the hurricane, the mean quarterly number of unique persons dialyzed in Puerto Rico was 2834 per quarter (95% CI, 2771-2897); afterwards it dropped to 261 (95% CI, −348 to −175; relative change, 9.2%). The percentage of persons who had 1 or more dialysis sessions outside of Puerto Rico in the next quarter following a previous dialysis in Puerto Rico was 7.1% before Hurricane Maria (95% CI, 4.8 to 9.3). There was a significant increase of 5.8 percentage points immediately after the hurricane (95% CI, 2.7 to 9.0). The 6-month mortality rate per person-quarter was 0.08 (95% CI, 0.08 to 0.09), and there was a nonsignificant increase in level of mortality rates and a nonsignificant decreasing trend in mortality rates.

**Conclusions and Relevance:**

The findings of this cross-sectional study suggest there was a significant increase in the number of people receiving dialysis outside of Puerto Rico after Hurricane Maria. However, no significant differences in mortality rates before and after the hurricane were found, which may reflect disaster emergency preparedness among dialysis facilities and the population with kidney failure, as well as efforts from other stakeholders.

## Key Points

**Question:**

Did migration and mortality change among people with kidney failure after Hurricane Maria in Puerto Rico?

**Findings:**

In this cross-sectional study of 11 652 patients with kidney failure who were receiving dialysis care in Puerto Rico, Hurricane Maria was associated with a significant increase in the number receiving dialysis outside of the island and a decline in those receiving dialysis in Puerto Rico. There was no evidence of increased mortality among patients in need of dialysis after Hurricane Maria.

**Meaning:**

These findings suggest that emergency preparedness strategies enabled the availability of dialysis and continuity of care for patients in need of dialysis after this extreme weather event.

## Teaser

This cross-sectional study of patients with kidney failure in Puerto Rico examines dialysis care, migration, and mortality rates before, during, and after Hurricane Maria.

## Introduction

On September 20, 2017, Hurricane Maria—one of the most destructive hurricanes in US history—made landfall in Puerto Rico with winds estimated to reach 150 miles per hour.^1^ Hurricane Maria caused widespread damages that totaled more than $100 billion, ranking it the third costliest hurricane in the US.^1,2,3^ The hurricane destroyed Puerto Rico’s electric grid, leading to one of the longest and most extensive power outages in US history^4^; more than 200 days were required to restore power to most residents.^5^ Rainfall of 15 to 20 inches brought catastrophic flooding across Puerto Rico^6,7^ that interrupted water and sanitation services for approximately 50% of residents.^8^ Years after the disaster, utility services remain unreliable in rural areas.^9,10^

Power outages and limited water supply can substantially affect hospitals and the health system. As described by multiple news outlets,^11,12,13,14,15^ some hospitals struggled to provide basic care; 1 month after the hurricane, only 9 of the 68 hospitals had power restored.^16^ Similar to hospitals, it appears that dialysis facilities lacked fuel for backup generators, access to clean water, and functional telephone and internet services.^17,18,19,20^ Some dialysis centers had to transport medical supplies, water filtration systems, generators, and other medical equipment from the mainland to their clinics in Puerto Rico.^21^ The disruption to the health care system may have affected populations that are particularly reliant on health care.^11^ For instance, people with kidney failure typically require specialized care, including regular dialysis, for survival.^22^ Anecdotal reports suggest that many persons with kidney failure left Puerto Rico or were evacuated to receive dialysis care before Hurricane Maria or in its aftermath, but empirical estimates of migration and outcomes for this population are unavailable.^23,24^

Patients with kidney failure have higher mortality rates and worse access to specialty care in Puerto Rico than in the US,^25^ and Hurricane Maria severely affected the health system.^26^ We assessed changes in migration and mortality among persons with kidney failure in Puerto Rico after Hurricane Maria and hypothesized that the hurricane was associated with increases in the overall level and the rate of both.

## Methods

The Brown University institutional review board and the US Centers for Medicare & Medicaid Services (CMS) Privacy Board approved the study protocol and waived the need for informed consent. The study followed the Strengthening the Reporting of Observational Studies in Epidemiology (STROBE) reporting guidelines.

### Study Design and Population

This cross-sectional study used an interrupted time-series (ITS) design to evaluate the number of unique persons receiving dialysis in each quarter, migration rates in the next quarter, and quarterly mortality rates within 6 month periods attributable to the hurricane by comparing outcomes *before* Hurricane Maria (before October 1, 2017) to outcomes *during* and *after* the hurricane (combined; ie, October 1, 2017-March 31, 2020). The during-hurricane period was defined as the fourth quarter 2017 (October 1-December 31, 2017) and after-hurricane period was defined as January 1, 2018, to March 31, 2020.

The study population comprised all patients with either prevalent or incident cases of kidney failure who received 1 dialysis session or more (hemodialysis and/or peritoneal dialysis) at a facility in Puerto Rico from the first quarter of 2012 through the first quarter of 2020. Therefore, any patient who received dialysis in Puerto Rico at any time during the study period and was then evacuated would be included in the analysis.

The Renal Management Information System (REMIS) database contains demographic information, diagnosis, treatment history,^27^ and facility location for all patients. The data include quarterly information for all patients (incident and prevalent) with kidney failure receiving maintenance dialysis, including the dialysis facility at which they received treatment. Facilities were identified using clinician-reported information. The study sample comprised 82 319 person-quarters and 11 652 unique individuals from 2012 to 2020.

### Study Measures

#### Main Exposure

The main exposure was during and after Hurricane Maria (October 1, 2017-March 31, 2020) vs before Hurricane Maria (January 1, 2012-October 1, 2017), measured in quarters.

#### Outcome Measures

First, we assessed the number of unique persons dialyzed in Puerto Rico each quarter. Second, for all patients receiving dialysis in a Puerto Rican facility in a given quarter, we calculated the percentage of persons who had previously received dialysis treatment in Puerto Rico and then received treatment at an outside facility (eg, facility on the US mainland) during the next quarter (ie, outside migration) using REMIS data. Of note, patients may or may not have changed residence. Third, we calculated 6-month mortality rates at the person-quarter level for people who had at least 1 dialysis event at a Puerto Rican facility.

Information regarding deaths was obtained from the End-Stage Renal Disease (ESRD) Death Notification (CMS 2746).^28^ Dialysis facilities performing maintenance dialysis are required to submit this completed form within 2 weeks of a patient’s date of death. To assess data accuracy, we examined whether the REMIS data captured the totality of dialysis care and deaths for our study population, including those who migrated or were receiving care immediately before or after Hurricane Maria. To do this, we calculated the proportion of all patients receiving dialysis in Puerto Rico in a quarter who we could not identify in the next 2 quarters as receiving maintenance dialysis, transplantation, or having died. There were no major differences before and after October 1, 2017 (more details are available in eFigure 1 in the Supplement).

#### Other Measures

Covariates from all the incident and prevalent cases among the study patients were obtained from the ESRD Medical Evidence Report (CMS 2728),^29^ which captures patient demographic information, including self-reported race and ethnicity, at the time of dialysis initiation. For prevalent cases, we used patient data from their corresponding CMS 2728 forms. We included the following variables in the study analyses: age, sex, race and ethnicity, health insurance coverage (at the time of dialysis initiation), and primary cause of kidney failure (diabetes, hypertension, or other).

### Statistical Analysis

First, we compared whether there were any differences regarding demographic characteristics and the primary cause of kidney failure among patients who were receiving dialysis in Puerto Rico before, during, and after the hurricane. These are not mutually exclusive groups—some patients were included in more than 1 group. The before hurricane group included patients with kidney failure receiving 1 or more dialysis treatments in Puerto Rico prior to Hurricane Maria (anytime from the first quarter of 2012 through the third quarter of 2017). The during hurricane group was comprised of patients receiving 1 or more dialysis sessions in Puerto Rico during the fourth quarter of 2017 (October-December) and when Puerto Rico was most affected by the hurricane. Finally, the last group (after the hurricane) included all patients with kidney failure who received dialysis care in Puerto Rico from January 2018 through March 2020. We compared the differences using analysis of variance and χ^2^ tests.

Second, we used an ITS design to evaluate the number of unique persons receiving dialysis in each quarter, migration rates in the next quarter, and mortality rates within 2 quarters attributable to the hurricane by comparing outcomes before Hurricane Maria (before October 1, 2017) with during and after Hurricane Maria (on/after October 1, 2017). Please see the equation in eTable 1 in the Supplement). The ITS approach is a quasi-experimental design that can evaluate the effect of the intervention or event while accounting for a secular trend; it has been used to understand similar phenomena.^30,31,32^ The ITS is modeled using segmented regression analysis where the time periods are divided into before and after intervention periods to estimate the before intervention level and slope and the changes in the level and slope from before to after intervention. Thus, in the ITS analysis, the before-hurricane period was comprised of 23 quarters (January 2012-September 2017), and the after-hurricane period spanned 10 quarters (October 2017-March 2020).

The segmented regression models with a first-order autoregressive errors included a linear time trend and quarter-fixed effects (separate dummy indicator variable for each quarter to account for seasonality). The segmented regression models with a first-order autoregressive errors included a linear time trend and quarter-fixed effects (separate dummy indicator variable for each quarter to account for seasonality), a binary indicator variable was set to 0 prior to the quarter during which Hurricane Maria occurred and set to 1 during and after that quarter (change in the outcome), an *interaction* term between time trend and the hurricane (linear trend and binary indicator to capture change in trend that occurs after).^31^ We also conducted stratified analysis by age and sex. In addition, we ran a Poisson model to estimate the number of unique persons receiving dialysis in each quarter as a sensitivity.

Data analyses were conducted using SAS, version 9.4 (SAS Institute Inc) February 12, 2019, to June 16, 2022. Tests were 2-tailed and statistical significance was defined as *P* = .05.

## Results

### Patient Characteristics

The total study sample comprised 11 652 unique persons (mean [SD] age, 59 [14.7] years; 7157 [61.6%] men and 4465 [38.4%] women; 10 675 [91.9%] Hispanic individuals; non-Hispanic individuals: 46 [3.0% ] Black, 476 [4.1%] White, and 124 [1.1%] of other race/ethnicity) who had received dialysis care at facilities in Puerto Rico at any time from 2012 to 2020 (first quarter). Among these, 9022 patients received dialysis care *before* Hurricane Maria; 2592 *during* the first quarter after the hurricane (October 1, 2017-December 31, 2017); and 5397 *after* the hurricane—the sum of these numbers exceeds 11 652 because some patients received dialysis during more than 1 period (Table 1). There were minor differences in age, Hispanic ethnicity, Medicaid coverage, and diabetes at dialysis initiation among the 3 group periods. In the during-hurricane period, patient age was younger at dialysis initiation than in the before- and after-hurricane periods (55.2 [95% CI, 54.6-55.7] vs 58.4 [95% CI, 58.1-58.7] vs 58.4 [95% CI, 58.0-58.7] years, respectively). Similarly, a higher proportion of patients self-reported Hispanic ethnicity during the hurricane (97.0%) compared with during the other periods (91.4% vs 95.1%, before and after Hurricane Maria, respectively). A slightly lower proportion of patients were receiving hemodialysis during the hurricane (87.2%) compared with the other periods (89.9% vs 89.6%, before and after Maria, respectively). In terms of health insurance, patients during the hurricane were 2% to 3% more likely to be enrolled in Medicaid at the time of dialysis initiation compared with the other patients before and after the hurricane.

**Table 1.** Patient Characteristics at Dialysis Initiation, Before, During, and After Hurricane Maria (82 319 person-quarters)

Characteristic	No. (%)				*P* value for comparisons^a^	
	All periods (Jan 1, 2012-Mar 31, 2020)	Before (Jan 1, 2012-Sep 30, 2017)	During (Oct 1, 2017-Dec 31, 2017)	After (Jan 1, 2018-Mar 31, 2020)	1	2
Unique persons, No.	11 652	9022	2592	5397	NA	NA
Age, mean (SD)^b^	59 (14.7)	58.4 (14.8)	55.2 (13.6)	58.4 (14.3)	<.001	<.001
Sex^b^						
Male	7157 (61.6)	5545 (61.6)	1622 (62.7)	3327 (61.8)	.41	.28
Female	4465 (38.4)	3457 (38.4)	966 (37.3)	2059 (38.2)		
Race and ethnicity^c^						
Hispanic	10675 (91.9)	8228 (91.4)	2510 (97.0)	5120 (95.1)	<.001	<.001
Non-Hispanic						
Black	346 (3.0)	292 (3.2)	Small cell	61 (1.1)		
White	476 (4.1)	361 (4.0)	55 (2.1)	178 (3.3)		
Other	124 (1.1)	120 (1.3)	Small cell	26 (0.5)		
Medical coverage						
Dual	783 (6.7)	583 (6.5)	139 (5.4)	373 (6.9)	<.001	<.001
Medicaid	1308 (11.2)	1006 (11.2)	370 (14.3)	672 (12.5)		
Medicare	1480 (12.7)	1126 (12.5)	279 (10.8)	665 (12.3)		
Other	8081 (69.4)	6307 (69.9)	1804 (69.6)	3687 (68.3)		
Dialysis type						
Hemodialysis^b^	10634 (90.2)	8114 (89.9)	2260 (87.2)	4838 (89.6)	<.001	<.001
Peritoneal	1074 (9.1)	838 (9.3)	321 (12.4)	538 (10.0)		
Primary cause of kidney failure						
Diabetes						
No	4803 (41.2)	3730 (41.3)	1151 (44.4)	2297 (42.6)	<.001	<.001
Yes	6849 (58.8)	5292 (58.7)	1441 (55.6)	3100 (57.4)		
Hypertension						
No	9676 (83.0)	7540 (83.6)	2138 (82.5)	4437 (82.2)	.03	.06
Yes	1976 (17.0)	1482 (16.4)	454 (17.5)	960 (17.8)		

^a^ *P* values for comparison 1: all patients in Puerto Rico during Hurricane Maria regardless of before or after; those who were in Puerto Rico only after or (before and after, not during); and those who were in Puerto Rico before only. *P* value for comparison 2: all patients who did not leave Puerto Rico after Hurricane Maria; left Puerto Rico during Maria; and in Puerto Rico only before. *P* values performed by either analysis of variance or χ^2^ test (exact); percentages are reported in parenthesis unless otherwise noted.

^b^ Variables contain a small proportion of missing values (≤2%).

^c^ The Centers for Medicare & Medicaid Services defines “Non-Hispanic Other” as respondents who are unable to identify with any of the race categories provided in form 2728.

### Migration and Mortality

**Figure 1.**
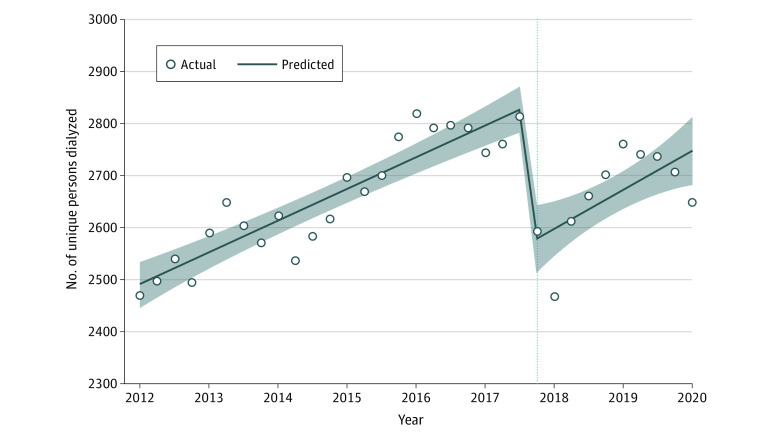
Estimated Changes in the Number of Unique Persons Undergoing Dialysis Treatment in Puerto Rico Before vs During and After Hurricane Maria The vertical dotted line represents the date of occurrence of Hurricane Maria; the shaded areas represent confidence limits.

The ITS segmented regression for the number of unique persons per quarter receiving dialysis care in Puerto Rico is shown in Figure 1 (full model results are available in Table 2). Before Hurricane Maria, the mean number of unique persons undergoing dialysis per quarter was 2834 (95% CI, 2771 to 2897). After Hurricane Maria, the mean number of unique persons undergoing dialysis dropped by 261 (95% CI, −348 to −175), a relative reduction of 9.2% (*P* <.001). There was no significant change in the trend of number of unique persons dialyzed after the hurricane (95% CI, −3 to 6; *P* = .58). When stratified by age or sex, adults younger than 65 years and men showed a larger reduction than their counterparts (results of the stratified analysis are available in eTable 2 in the Supplement). We also ran a Poisson model as a sensitivity analysis and the results were similar to those of the main specification (eTable 3 in the Supplement).

**Table 2.** Adjusted Changes in the Number of Unique Patients Receiving Dialysis, Proportion Who Migrated in the Next Quarter, and 6-Month Mortality Rate After Hurricane Maria

Period^a^ and change	Estimate (95% CI)
Unique persons dialyzed per quarter in Puerto Rico, No.	
Baseline level before hurricane	2834 (2771 to 2897)
Level change after hurricane	−261 (−348 to −175)
Baseline trend	5 (4 to 6)
Trend change after hurricane	1 (−3 to 6)
People with ≥1 dialysis outside of Puerto Rico in the next quarter, %	
Baseline level before hurricane	7.1 (4.8 to 9.3)
Level change after hurricane	5.8 (2.7 to 9.0)
Baseline trend	0
Trend change after hurricane	−0.3 (−0.4 to −0.1)
Mortality rates (per person per quarter) in the next 6 months	
Baseline level before hurricane	0.08 (0.08 to 0.09)
Level change after hurricane	0
Baseline trend	0
Trend change after hurricane	0

^a^ The period before the hurricane was composed of 23 quarters (January 2012-September 2017); the period after the hurricane, 10 quarters (October 2017-March 2020).

**Figure 2.**
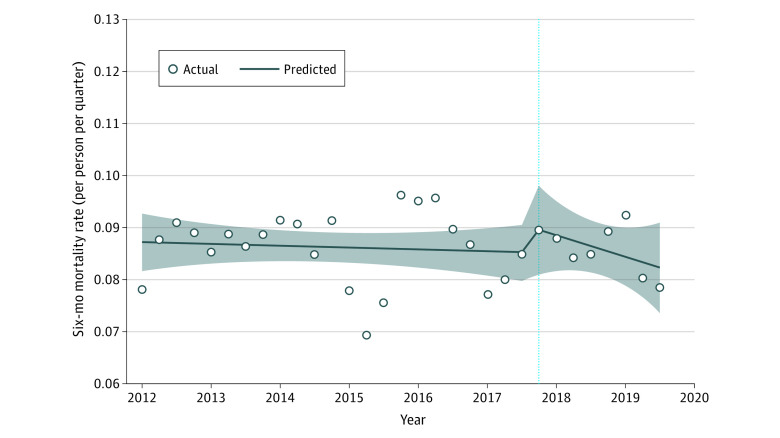
Estimated Changes in 6-Month Mortality Rates (per Person per Quarter) Among Patients With Kidney Failure Before vs During and After Hurricane Maria The vertical dotted line represents the date of occurrence of Hurricane Maria; the shaded areas represent confidence limits.

Figure 2 shows that the percentage of persons who had 1 or more dialysis sessions outside of Puerto Rico in the next quarter following dialysis in Puerto Rico was 7.1% before the hurricane (95% CI, 4.8 to 9.3). There was a significant increase of 5.8 percentage points (pp) immediately after the hurricane (95% CI, 2.7 to 9.0), or a relative change of 82%. There was a significant decrease in the trend of those who had 1 or more dialysis sessions outside of Puerto Rico by approximately 0.3 pp thereafter (95% CI, −0.4 to −0.1). Of note, the most common destinations for patients with kidney failure from Puerto Rico after the hurricane were Florida, Pennsylvania, Texas, Massachusetts, and New York (in order of most to least; results not shown because of small cell count). Similar to the main analysis, there was a significant increase in the percentage of people with dialysis outside of Puerto Rico of 5.3 pp (95% CI, 2.5 to 8.0) among adults younger than 65 years but nonsignificant among those 65 years and older (3.5 pp; 95% CI, −0.8 to 7.7). After the hurricane, there was a significant decrease in the trends for both groups (−0.3% and −0.3%, respectively). Results remained consistent when stratified by sex, with an increase of 5.1 pp (95% CI, 1.4 to 8.8) for women and 4.5 pp (95% CI, 1.8 to 7.6) for men right after the hurricane, with a decrease of 0.3% and 0.3% after hurricane trends, respectively (results of the stratified analysis are available in eTable 4 in the Supplement).

**Figure 3.**
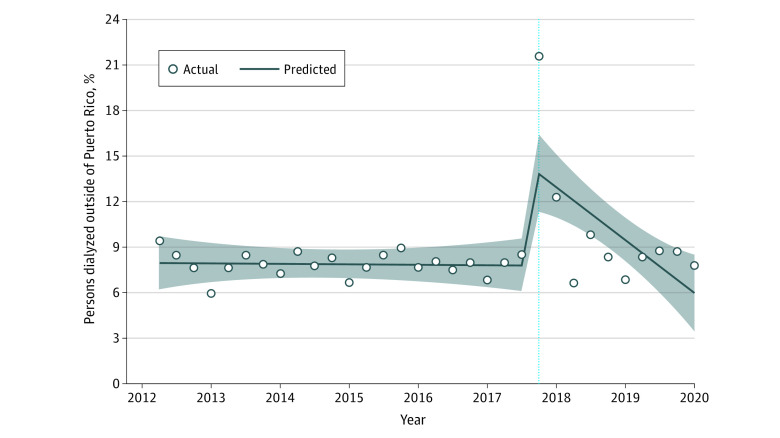
Estimated Changes in the Percent of Persons Undergoing Dialysis Treatment Outside of Puerto Rico Before vs During and After Hurricane Maria The vertical dotted line represents the date of occurrence of Hurricane Maria; the shaded areas represent confidence limits.

Figure 3 illustrates the ITS segmented regression for 6-month mortality rates. Mean mortality rates per person per quarter were 0.08 (95% CI, 0.08 to 0.09) before the hurricane. There was a nonsignificant increase in level of mortality rates of 0.00 pp (95% CI, −0.01 to 0.00; *P* =.59) and a nonsignificant decreasing trend in mortality rates of 0.00 pp (95% CI, 0.00 to 0.00; *P* =.61). Results remained consistent after stratifying by age and sex (eTable 5 in the Supplement).

## Discussion

Hurricane Maria has been described as one of the worst natural disasters in US history.^23^ In this study of people with kidney failure, we found that there were 2592 unique patients receiving dialysis in Puerto Rico during Hurricane Maria. However, there were substantial differences in the number of unique persons undergoing dialysis in Puerto Rico and the percentage of people who received dialysis at a different facility outside of Puerto Rico immediately after Maria, indicating migration among people with kidney failure to the mainland. The destination states for migration were comparable with the overall migration trends after the hurricane from the general Puerto Rican population.^33^ Strikingly, there were no significant differences in the mortality rate or trend among patients receiving dialysis care before and after the hurricane.

These results are consistent with prior studies that have found that hurricanes that cause severe damage and destroy the built environment often result in temporary displacement and sometimes permanent out-migration.^34,35^ After Hurricane Katrina in 2005, a large number of patients with kidney failure left affected areas or were displaced and received dialysis at facilities in nonaffected areas in Georgia, Louisiana, and Texas.^36^ In addition, other studies show the associations of these natural disasters with treatment and health outcomes. For example, approximately 50% of patients with kidney failure living in the area affected by Hurricane Katrina reported missing 1 or more hemodialysis sessions—resulting in a 5- to 7-fold increase in missed sessions after that hurricane.^37^ These missed dialysis sessions were associated with hypertensive emergencies, respiratory failure, and other complications that required hospitalization.^36,37^ In addition, there has been some evidence that rates of depression and posttraumatic stress disorder among patients with kidney failure trended upwards after Hurricane Katrina, potentially disrupting disease management.^38^ After Hurricane Sandy in 2012, there were higher rates of hospitalizations, emergency department visits, and mortality among patients with kidney failure.^39^ This appeared not to be the case among people with kidney failure in Puerto Rico, which was not consistent with our hypothesis. Although we may never know the precise number of deaths directly and indirectly associated with Hurricane Maria, scientific estimates range from 910 to 4645 deaths, although most estimates are closer to the lower end of this range.^40^

The finding of unchanged mortality rates among patients with kidney failure in Puerto Rico may be partially explained by improvements in disaster planning among dialysis facilities and clinicians, which has been informed by prior experiences with other disasters.^41^ Disaster planning and preparation for health services have gained importance in the wake of climate change (eg, the Kidney Community Emergency Response Program).^42^ Because large-scale disasters are likely to interrupt dialysis treatment and care that are needed by patients with complex conditions,^36^ government officials and stakeholders have made efforts to prioritize the population with kidney failure and maintain continuity of dialysis treatment. Our findings suggest these efforts may have been successful.

The CMS issued a statement on October 17, 2017, that indicated that people with kidney failure in Puerto Rico were a top priority population.^20^ The US Department of Health and Human Services along with the Federal Emergency Management Agency (FEMA) delivered water, fuel, replacement generators, and necessary supplies to dialysis centers and hospitals to help them remain operational.^43^ The US Government Accountability Office stated that Hurricane Maria brought on a “large federal disaster response,” helping to evacuate dialysis patients.^44^ A large manager of dialysis centers in Puerto Rico and the US has described its experience and efforts (mobilizing staff and maintenance personnel) to get facilities up and running and to care for patients who were waiting outside of their doors after Hurricane Maria.^19^ According to FEMA reports, by November 10, 2017, about 94% of dialysis centers in Puerto Rico were fully operational and open to treat patients.^45^ This appears to be consistent with our data. According to the REMIS data, only 4 of 59 facilities permanently closed after Hurricane Maria, and those facilities served approximately 50 to 55 patients (eFigure 2 and eFigure 3 in the Supplement).

The CMS published a disaster preparedness guide tailored to dialysis facilities in 2011 with best practices for preparing for extreme events, which may have been partially implemented in Puerto Rico.^23,46^ This preparedness guide states that dialysis facilities should ensure the availability of “dialysis care and to expedite resumption of dialysis operations.”^23,46^ Yet according to the nephrology community, there is still “a lot to learn” from these hurricanes.^47^ It is clear that advance preparations for staff and patients are essential (eg, clean drinking water, medical supplies, medications, fuel, food).^23,47^ Some dialysis centers have disaster-planning information available for patients and/or family members on their websites, and some centers have formed disaster response teams.^48,49^ An emergency preparedness checklist includes having a functional generator and water tank; staff who are available on-call; a network of dialysis centers as a contingency plan; a directory of patients; a 2-week supply of medications; a preemptive dialysis treatment before the event; and preemptive admission for patients who reside in vulnerable areas. A complete list has been published by Bonilla-Felix and Suarez-Rivera.^23^ In addition, some facilities have decided to increase the number of personal generators.^19^ Involvement among the government and stakeholders will continue to be critical to providing access to dialysis services and continuous treatment for people with kidney failure in areas affected by natural disasters.

The findings of this study have research and policy implications. First, more than 60% of Puerto Rico residents, most of whom are enrolled in Medicare, rely on managed care plans to receive health care on the island^50,51,52^; these plans often require subscribers to use in-network practitioners and facilities and to obtain prior approvals. A recent case report determined that patients missed dialysis sessions because insurance coverage did not transfer to the US mainland and switching or disenrolling was not a straightforward process.^53^ Therefore, Medicare enrollees in Puerto Rico who migrate to the mainland US may face barriers accessing care, at least in the short term.

Second, although there was no significant increase in mortality rates among people with kidney failure after Hurricane Maria, mortality rates among patients with kidney failure remained high in Puerto Rico compared with the US mainland.^25^ In 2017, the mortality rate per 1000 persons per year was 168.8 in Puerto Rico compared with 135.9 in the US as a whole.^54^ Based on the experience with Hurricane Maria, the Government Accountability Office has recommended that the Department of Health and Human Services develop strategies with other government agencies, including the development of a response team, a system to track patients who are evacuated through the National Disaster Medical System, and a plan to provide care to patients with chronic conditions who live in remote communities.^55^ These recommendations are applicable to the kidney failure population. However, the health care system in Puerto Rico has faced a number of ongoing challenges, including low funding and shortages of health care facilities and medical professionals.^56,57^ In addition to patients, health care practitioners are continuing to migrate away from Puerto Rico.^58,59,60^ The exodus appears to be accelerating after Hurricane Maria, with health care professionals leaving for the US mainland for better salaries and resources. By 2019, the island had lost about 15 % of its medical specialists, according to data from Puerto Rico’s College of Physicians and Surgeons, leaving fewer than 11 000 specialists to serve Puerto Rico’s population of 3.2 million.^14^ Currently, some reports have documented a 4 to 6 month waiting period for a consultation with a specialist such as a nephrologist.^61^ Physicians describe this as not only a problem for the health care system, but also as a humanitarian crisis.^62^ Thus, it is crucial to evaluate the current needs of the health care system in Puerto Rico and to understand the long-term consequences of Hurricane Maria among this population.

### Limitations

Our study had some limitations. First, Hurricane Maria caused widespread damage across Puerto Rico, and thus, we are unable to include a concurrent control group of people in Puerto Rico who were not exposed to the hurricane. However, ITS models can evaluate the effect of the intervention or event while accounting for a secular trend, and they do not require a concurrent control group.^30,31,32^ Second, REMIS data are generated quarterly. Therefore, although we are unable to use the exact date on which Hurricane Maria occurred in Puerto Rico, the beginning of the fourth quarter of 2017 (October 1, 2017) approximately corresponds to the date of the hurricane’s landfall. Furthermore, the aftermath of Hurricane Maria lasted for months because the island lost power and electricity, which are essential services for people receiving dialysis.^11,12,13,14,15^ Lastly, demographic and clinical characteristics were measured or reported at dialysis initiation. We lack certain time-varying covariates, such as number of chronic conditions and/or insurance changes, which may moderate these outcomes. However, there appeared to be only minor differences across the different groups at dialysis initiation.

## Conclusions

This cross-sectional study among patients with kidney failure in Puerto Rico before and after Hurricane Maria showed a significant increase in the number of patients receiving dialysis outside of Puerto Rico after the hurricane, as well as a significant decline in the number of unique persons receiving dialysis in Puerto Rico, indicating a substantial migration after Hurricane Maria. Contrary to the findings of other studies and reports for the general population, we did not find evidence of increased mortality among patients with kidney failure after Hurricane Maria. Our study findings may reflect the disaster emergency preparedness of individual facilities and/or other organizations to ensure availability of dialysis care and limit the number of missed dialysis sessions and/or hospitalizations among patients being treated with dialysis in Puerto Rico.^40,42,46,48,63,64,65^ Some of the strategies used in Puerto Rico may be applicable to other vulnerable populations to avoid hospitalization and mortality after natural disasters.

## Notes

Supplement. eFigure 1. Patients who are alive but did not receive dialysis care in the next 6 months eTable 1. Interrupted time series equation eTable 2. Interrupted time series full model results of number of unique persons dialyzed per quarter in Puerto Rico, stratified by age and sex eTable 3. Interrupted time series model results with Poisson distribution of number of unique persons dialyzed per quarter in Puerto Rico eTable 4. Interrupted time series full model results of percentage of people who had at least 1 dialysis outside of Puerto Rico in the next quarter, stratified by age and sex eTable 5. Interrupted time series full model results of mortality rates within the next 2 quarters, stratified by age and sex eFigure 2. Number of dialysis facilities in Puerto Rico eFigure 3. Number of patients dialyzed in facilities that permanently closed in Puerto Rico
