# Supplementary material for: Changes in Migration and Mortality Among Patients With Kidney Failure in Puerto Rico After Hurricane Maria
Source: JAMA Health Forum. 2022 Aug 12;3(8):e222534. doi: 10.1001/jamahealthforum.2022.2534 (PMC9375170; doi:10.1001/jamahealthforum.2022.2534)
Supplement: Supplement. — eFigure 1. Patients who are alive but did not receive dialysis care in the next 6 months eTable 1. Interrupted time series equation eTable 2. Interrupted time series full model results of number of unique persons dialyzed per quarter in Puerto Rico, stratified by age and sex eTable 3. Interrupted time series model results with Poisson distribution of number of unique persons dialyzed per quarter in Puerto Rico eTable 4. Interrupted time series full model results of percentage of people who had at least 1 dialysis outside of Puerto Rico in the next quarter, stratified by age and sex eTable 5. Interrupted time series full model results of mortality rates within the next 2 quarters, stratified by age and sex eFigure 2. Number of dialysis facilities in Puerto Rico eFigure 3. Number of patients dialyzed in facilities that permanently closed in Puerto Rico [file jamahealthforum-e222534-s001.pdf]

Supplemental Online Content

Rivera-Hernandez M, Kim D, Nguyen KH, et al. Changes in migration and mortality among patients with kidney failure in Puerto Rico after Hurricane Maria. *JAMA Health Forum.* 2022;3(8):e222534. doi:10.1001/jamahealthforum.2022.2534

eFigure 1. Patients who are alive but did not receive dialysis care in the next 6 months

eTable 1. Interrupted time series equation

eTable 2. Interrupted time series full model results of number of unique persons dialyzed per quarter in Puerto Rico, stratified by age and sex

eTable 3. Interrupted time series model results with Poisson distribution of number of unique persons dialyzed per quarter in Puerto Rico

eTable 4. Interrupted time series full model results of Percentage of people who had at least 1 dialysis outside of Puerto Rico in the next quarter, stratified by age and sex

eTable 5. Interrupted time series full model results of mortality rates within the next 2 quarters, stratified by age and sex

eFigure 2. Number of dialysis facilities in Puerto Rico

eFigure 3. Number of patients dialyzed in facilities that permanently closed in Puerto Rico

This supplemental material has been provided by the authors to give readers additional information about their work.

© 2022 Rivera-Hernandez et al. *JAMA Health Forum*

eFigure1. Patients who are alive but did not receive dialysis care in the next six months

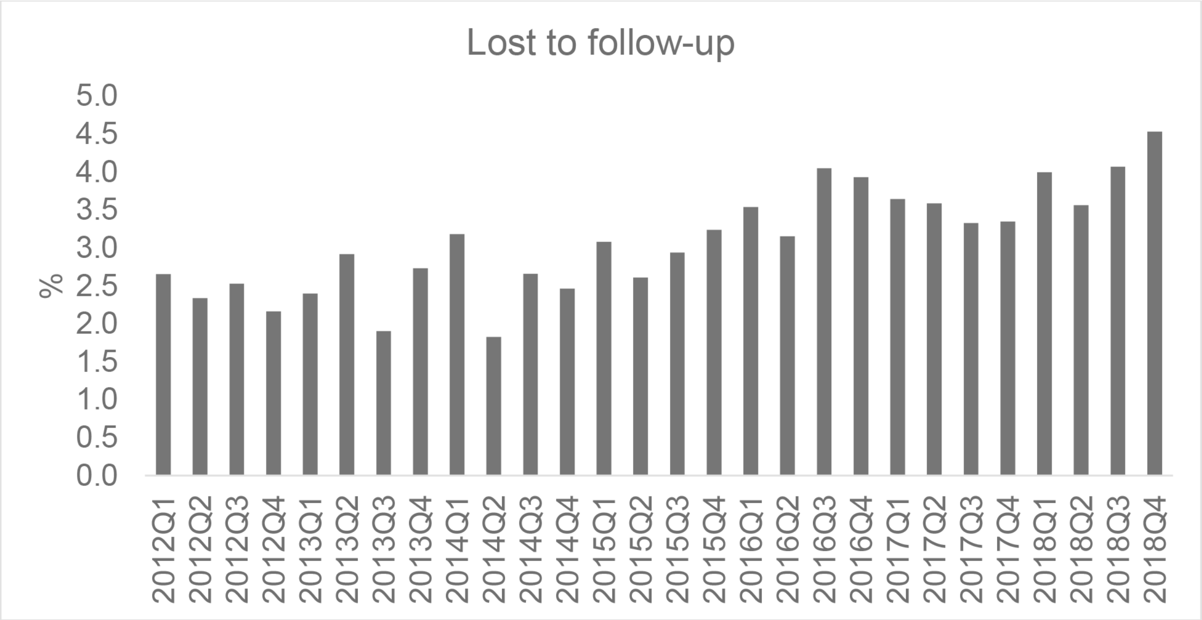

Note: These patients were alive, but did not receive dialysis treatments within the next six months (excluding those who received a transplant)

© 2022 Rivera-Hernandez et al. *JAMA Health Forum*

eTable 1. Interrupted time series equation

$$Y_t = \beta_0 + \beta_1 Time + \beta_2 PostMaria_t + \beta_3 Time\ x\ PostMaria_t + \beta_4 Quarter\ \{Quarter = 2\} + \beta_5 Quarter\ \{Quarter = 3\} + \beta_6 Quarter\ \{Quarter = 4\} + \varepsilon_t$$

Where Y_t is the outcome (1: number of unique persons dialyzed; 2: rate of 6-month mortality within 6 months; and 3: proportion of persons who switched facilities per person per quarter) at a given quarter (*t*), the *Time* variable represents a linear time trend, the *PostMaria* variable indicates whether the time *t* is before (0) or after (1) Hurricane Maria, the *Time x PostMaria* variable represents time elapsed since Hurricane Maria, taking a value of 0 prior to Maria, {Quarter=2},{Quarter=3} and {Quarter=4} are dummy variables for each quarter to account for seasonality, and the error term (ε_t) is assumed to follow a first-order autoregressive (AR) process, AR(1). The coefficients β_2 and β_3 are the parameters of interest. The coefficient β_2 measures a change in level in migration that occurs immediately following Maria and $\beta_3$is the change in trend that occurs after Maria.

© 2022 Rivera-Hernandez et al. *JAMA Health Forum*

eTable 2. Interrupted time series full model results of number of unique persons dialyzed per quarter in Puerto Rico, stratified by age and sex

	Estimate	95% Confidence Interval
Stratified (Age <65)		
Baseline level before Maria	2197	(2165 – 2229)
Level change after Maria	-166	(-220 – -113)
Baseline trend	3	(3 – 4)
Trend change after Maria	-1	(-3 – 2)
Stratified (Age =>65)		
Baseline level before Maria	644	(619 – 668)
Level change after Maria	-96	(-138 – -55)
Baseline trend	2	(1 – 2)
Trend change after Maria	2	(0 – 4)
Stratified (Female)		
Baseline level before Maria	1071	(1047 – 1095)
Level change after Maria	-102	(-142 – -62)
Baseline trend	1	(1 – 2)
Trend change after Maria	2	(0 – 4)
Stratified (Male)`		
Baseline level before Maria	1767	(1740 – 1794)
Level change after Maria	-163	(-208 – -118)
Baseline trend	4	(3 – 5)
Trend change after Maria	-1	(-3 – 2)

Note: The pre-Maria period was comprised of 23 quarters (January 2012 to September 2017), and the post-Maria period spanned ten quarters (October 2017 to March 2020).

© 2022 Rivera-Hernandez et al. *JAMA Health Forum*

eTable 3. Interrupted time series model results with Poisson distribution of number of unique persons dialyzed per quarter in Puerto Rico

	Estimate	95% Confidence Interval
Baseline level before Maria	8.0	(7.9 – 8.0)
Level change after Maria	-0.1	(-0.1 – -0.1)
Baseline trend	0.0	(0.0 – 0.0)
Trend change after Maria	0.0	(0.0 – 0.0)

Note: The pre-Maria period was comprised of 23 quarters (January 2012 to September 2017), and the post-Maria period spanned ten quarters (October 2017 to March 2020).

eTable 4. Interrupted time series full model results of percentage of people who had at least one dialysis outside of Puerto Rico in the next quarter, stratified by age and sex

	Estimate	95% Confidence Interval
Stratified (Age <65)		
Baseline level before Maria	7.1	(5.2 – 9.0)
Level change after Maria	5.3	(2.5 – 8.0)
Baseline trend	0.0	(0.0 – 0.0)
Trend change after Maria	-0.3	(-0.4 – -0.1)
Stratified (Age =>65)		
Baseline level before Maria	9.2	(6.3 – 12.1)
Level change after Maria	3.5	(-0.8 – 7.7)
Baseline trend	0.0	(0.0 – 0.1)
Trend change after Maria	-0.3	(-0.6 – -0.1)
Stratified (Female)		
Baseline level before Maria	7.8	(5.2 – 10.3)
Level change after Maria	5.3	(1.5 – 9.0)
Baseline trend	0.0	(0.0 – 0.1)
Trend change after Maria	-0.3	(-0.5 – -0.1)
Stratified (Male)		
Baseline level before Maria	7.4	(5.6 – 9.2)
Level change after Maria	4.6	(2.0 – 7.3)
Baseline trend	0.0	(0.0 – 0.0)
Trend change after Maria	-0.3	(-0.4 – -0.1)

Note: The pre-Maria period was comprised of 23 quarters (January 2012 to September 2017), and the post-Maria period spanned ten quarters (October 2017 to March 2020).

© 2022 Rivera-Hernandez et al. *JAMA Health Forum*

eTable 5. Interrupted time series full model results of mortality rates per person per quarter within the next six months, stratified by age and sex

	Estimate	95% Confidence Interval
Stratified (Age <65)		
Baseline level before Maria	0.08	0.1 – 0.1
Level change after Maria	0.03	0.0 – 0.0
Baseline trend	0.0	0.0 – 0.0
Trend change after Maria	0.0	0.0 – 0.0
Stratified (Age =>65)		
Baseline level before Maria	0.16	0.1 – 0.2
Level change after Maria	0.06	0.0 – 0.0
Baseline trend	0.0	0.0 – 0.0
Trend change after Maria	0.0	0.0 – 0.0
Stratified (Female)		
Baseline level before Maria	0.09	0.1 – 0.1
Level change after Maria	-0.06	0.0 – 0.0
Baseline trend	0.0	0.0 – 0.0
Trend change after Maria	0.0	0.0 – 0.0
Stratified (Male)		
Baseline level before Maria	0.09	0.1 – 0.1
Level change after Maria	0.009	0.0 – 0.0
Baseline trend	0.0	0.0 – 0.0
Trend change after Maria	0.0	0.0 – 0.0

Note: The pre-Maria period was comprised of 23 quarters (January 2012 to September 2017), and the post-Maria period spanned ten quarters (October 2017 to March 2020).

© 2022 Rivera-Hernandez et al. *JAMA Health Forum*

eFigure2. Number of dialysis facilities in Puerto Rico that were open, temporary closed and permanently closed in 2017

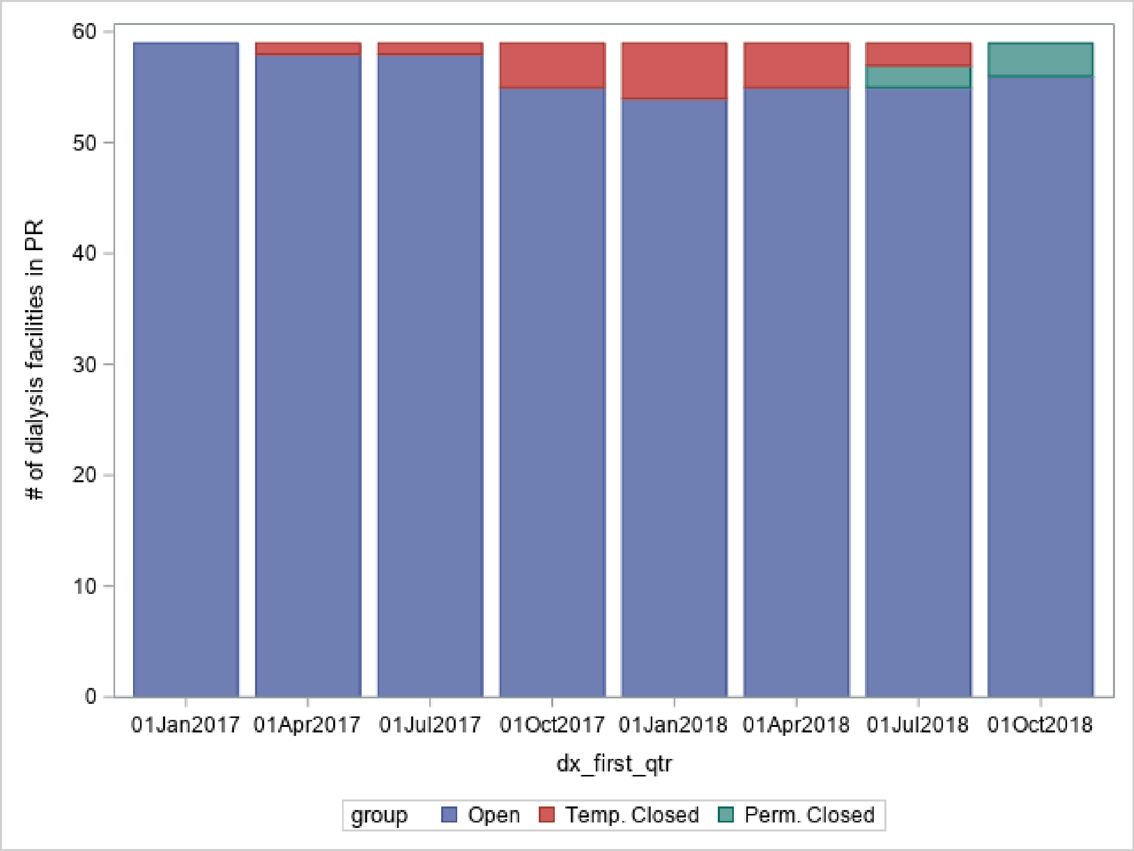

Note: Hurricane Maria was characterized using the fourth quarter in 2017 (10/01/2017)

© 2022 Rivera-Hernandez et al. *JAMA Health Forum*

eFigure3. Number of patients dialyzed in facilities that permanently closed in Puerto Rico

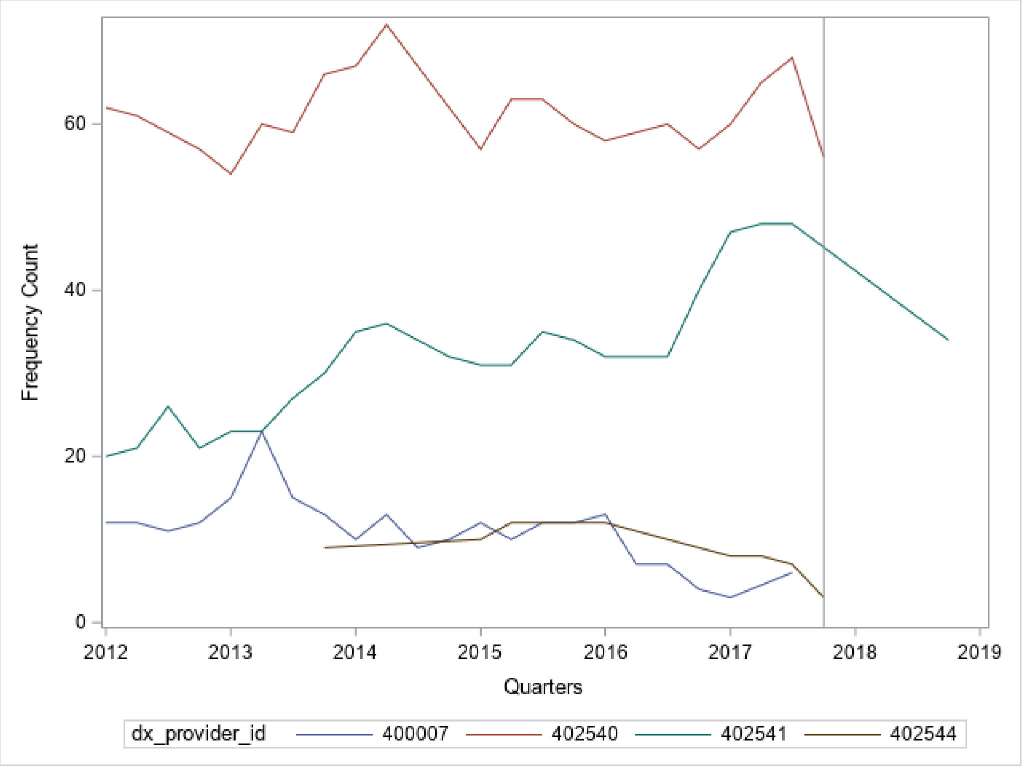

Note: Hurricane Maria was characterized using the fourth quarter in 2017 (10/01/2017)

© 2022 Rivera-Hernandez et al. *JAMA Health Forum*
